# Supplementary material for: MeJA Elicitation on Flavonoid Biosynthesis and Gene Expression in the Hairy Roots of Glycyrrhiza glabra L
Source: Genes (Basel). 2025 Nov 18;16(11):1387. doi: 10.3390/genes16111387 (PMC12652217; doi:10.3390/genes16111387)
Supplement: Supplementary file 1 [file genes-16-01387-s001.zip › genes-3973358-supplementary/Supplementary Figures.pdf]

Supplementary Files

MeJA elicitation on flavonoid biosynthesis and gene expression in the hairy roots of *Glycyrrhiza glabra* L.

Yutao Zhu[1*], Bohan Wang[1], Bingyi Xue[1], Runqian Wang[1], Ganlin Tang[1], Tao Zhu[1], Mei Zhao[1], Taotao Li[1], Chunli Liao[1], Huamin Zhang[1], Dongxiao Liu[1], Jianhua Chen[2], and Lianzhe Wang[1*]

[1] College of Life Science and Engineering, Henan University of Urban Construction, Pingdingshan, 467036, China

[2] Pingdingshan Academy of Agricultural Sciences, Pingdingshan, 467003, China

*** Correspondence:** Yutao Zhu, zyt17494@163.com; Lianzhe Wang, jijy99@126.com

1 Supplementary Figures and Tables

1.1 Supplementary Figures

A

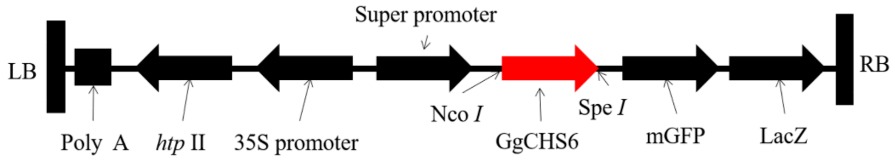

B

```
         10        20        30        40        50        60
ATGGTGAGTGTAGCTGAAATTCGCAAAGCTCAAAGGGCAGAAGGCCCTGCAAACATCTTG
 M  V  S  V  A  E  I  R  K  A  Q  R  A  E  G  P  A  N  I  L

         70        80        90       100       110       120
GCCATTGGTACTGCAAATCCACCAAATTGTGTTGATCAAAGTACTTATCCTGATTTTTAC
 A  I  G  T  A  N  P  P  N  C  V  D  Q  S  T  Y  P  D  F  Y

        130       140       150       160       170       180
TTTAAGATCACAAACAGTGAGCACAAGACCGAGCTTAAGGAAAAATTTCAGCGCATGTGT
 F  K  I  T  N  S  E  H  K  T  E  L  K  E  K  F  Q  R  M  C

        190       200       210       220       230       240
GATAAATCTATGATCAAGAAGCGATATATGTACCTAACGGAAGAGATTTTGAAAGAGAAT
 D  K  S  M  I  K  K  R  Y  M  Y  L  T  E  E  I  L  K  E  N

        250       260       270       280       290       300
CCTAACATTTGCGCTTATATGGCACCTTCTTTGGACGCTAGGCAAGACATGGTGGTCGTA
 P  N  I  C  A  Y  M  A  P  S  L  D  A  R  Q  D  M  V  V  V

        310       320       330       340       350       360
GAGGTGCCTAGACTAGGGAAGGAAGCTGCGGTCAAGCTATAAAAGAATGGGGCCAACCAA
 E  V  P  R  L  G  K  E  A  A  V  K  L  *  K  N  G  A  N  Q

        370       380       390       400       410       420
AGTCAAAGATCACCCACTTAATTTTTTGCACTACTAGGGTGTGGACATGCCGGCGCTGAT
 S  Q  R  S  P  T  *  F  F  A  L  L  G  C  G  H  A  G  A  D

        430       440       450       460       470       480
TACCAACTTACTAAACTCTTGGGTCTTCGCCCATATGTGAAAAGGTATATGATGTACCAG
 Y  Q  L  T  K  L  L  G  L  R  P  Y  V  K  R  Y  M  M  Y  Q

        490       500       510       520       530       540
CAAGGGTGTTTTGCAGGTGGCACGGTGCTTCGCTTGGCCAAAGACTTGGCGGAGAACAAC
 Q  G  C  F  A  G  G  T  V  L  R  L  A  K  D  L  A  E  N  N

        550       560       570       580       590       600
AAAGGTGCTCGTGTGCTAGTTGTTTGTTCTGAAGTTACTGCAGTCACATTCCGTGGCCCT
 K  G  A  R  V  L  V  V  C  S  E  V  T  A  V  T  F  R  G  P

        610       620       630       640       650       660
ACTGATACTCACCTAGATAGCCTTGTGGGACAAGCATTATTTGGAGATGGAGCAGCTGCA
 T  D  T  H  L  D  S  L  V  G  Q  A  L  F  G  D  G  A  A  A

        670       680       690       700       710       720
GTCATTGTTGGTTCTGACCCAATACCCGAAATTGAGAAGCCTATATTTGAGTTGGTTTGG
 V  I  V  G  S  D  P  I  P  E  I  E  K  P  I  F  E  L  V  W

        730       740       750       760       770       780
ACGGCACAAACAATAGCTCCAGATAGTGAAGGAGCCATTGATGGTCACCTTCGTGAAGTT
 T  A  Q  T  I  A  P  D  S  E  G  A  I  D  G  H  L  R  E  V

        790       800       810       820       830       840
GGGCTCACATTTCATCTTCTTAAAGATGTTCCCGGGATGTCTCAAAGAACATTGATAAAG
 G  L  T  F  H  L  L  K  D  V  P  G  M  S  Q  R  T  L  I  K
        850       860       870       880       890       900
CACTGACTGAGGCATTCCAACCATTAGGCATTCTGATTACAACTCAATCTTTTGGATTGC
 H  *  L  R  H  S  N  H  *  A  F  *  L  Q  L  N  L  L  D  C

        910       920       930       940       950       960
ACACCCAGGTGGACCGCAATTCTTGACCAAGTTGAGCAAAAGTTAGCTTTGAAACCTGAA
 T  P  R  W  T  A  I  L  D  Q  V  E  Q  K  L  A  L  K  P  E

        970       980       990      1000      1010      1020
AAGATGAAGGCCACTAGGGATGTGCTTAGTGATTATGGTAATATGTCAAGTGCATGTGTT
 K  M  K  A  T  R  D  V  L  S  D  Y  G  N  M  S  S  A  C  V

       1030      1040      1050      1060      1070      1080
CTATTCATCTTGGATGAGATGAGAAAGAAATCAGCTCAAAATGGACTTAAGACCACTGGC
 L  F  I  L  D  E  M  R  K  K  S  A  Q  N  G  L  K  T  T  G

       1090      1100      1110      1120      1130      1140
GAAGGACTCGAATGGGGTGTATTATTCGGCTTTGGACCTGGACTTACCATCGAAACTGTT
 E  G  L  E  W  G  V  L  F  G  F  G  P  G  L  T  I  E  T  V

       1150      1160      1170
GTTTTGCACAGTGTGGCTATATGA
 V  L  H  S  V  A  I  *
```

Supplementary Figure S1.

Construction of *GgCHS6* overexpression vector. **(A)** Schematic diagram of *GgCHS6* overexpression vector. **(B)** The DNA sequence of the *GgCHS6* and the sequence of the encoded protein.

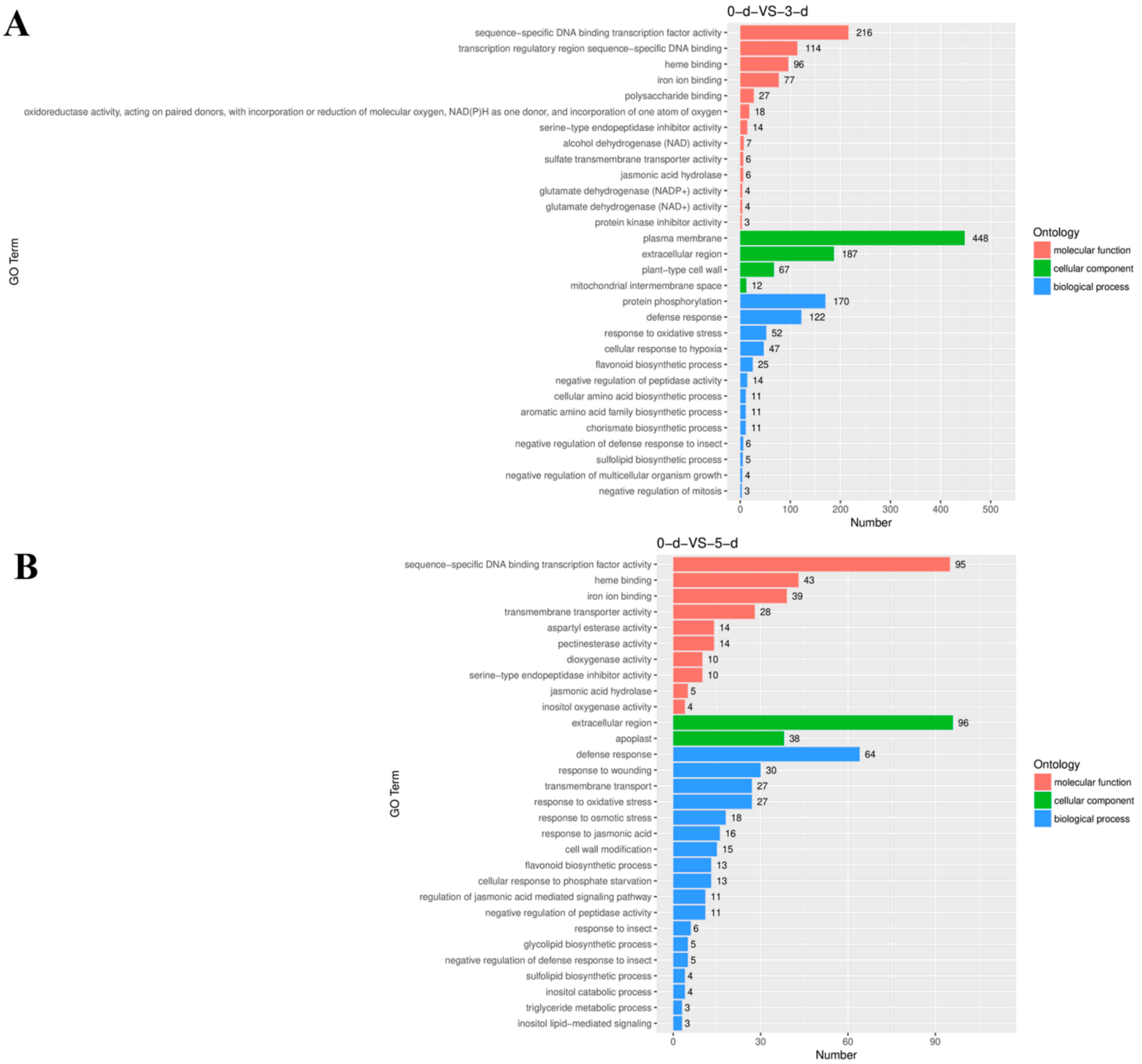

Supplementary Figure S2.

Gene Ontology (GO) functional classification analysis of DEGs in 0 vs. 3 d (A) and 0 vs. 5 d (B) based on RNA-Seq data. Based on sequence homology, all differentially expressed genes could be categorized into three main categories: molecular function, cellular component, and biological process.

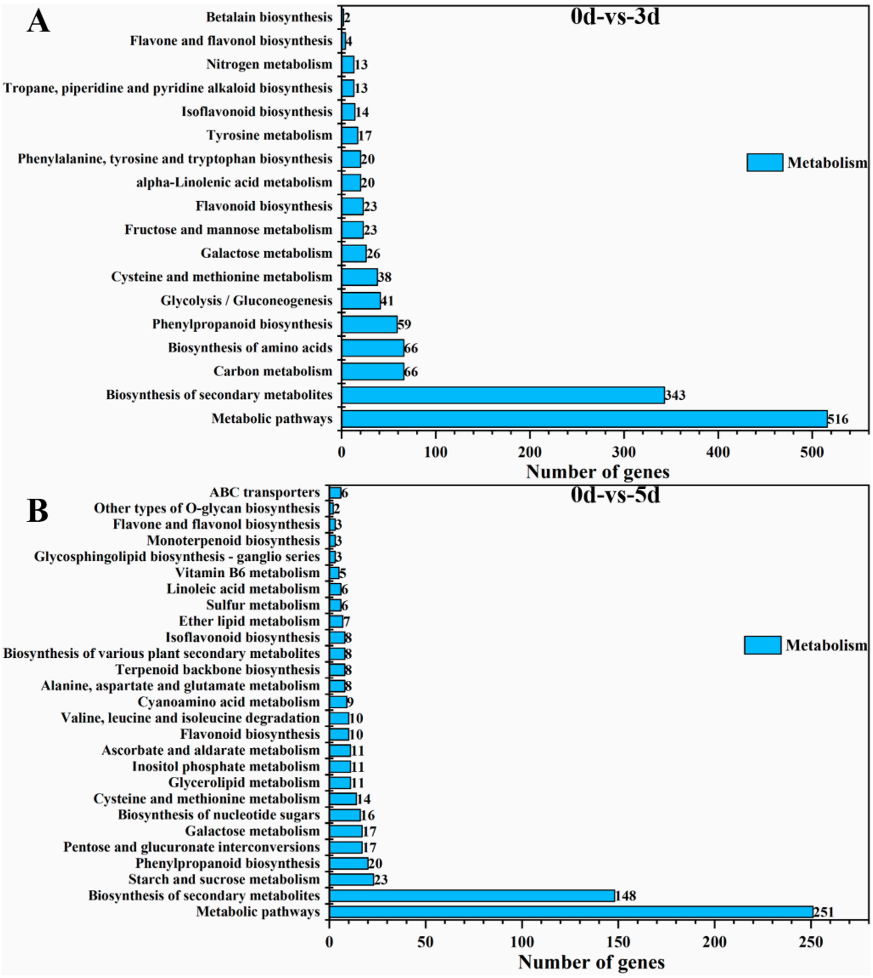

Supplementary Figure S3.

After 3 d and 5 d of MeJA treatment, the following KEGG pathways were mostly enriched in “metabolic pathways (ko01100)” (516 DEGs at 3 d and 251 DEGs at 5 d, respectively) and “biosynthesis of secondary metabolites (ko01110)” (343 DEGs at 3 d and 148 DEGs at 5 d, respectively).

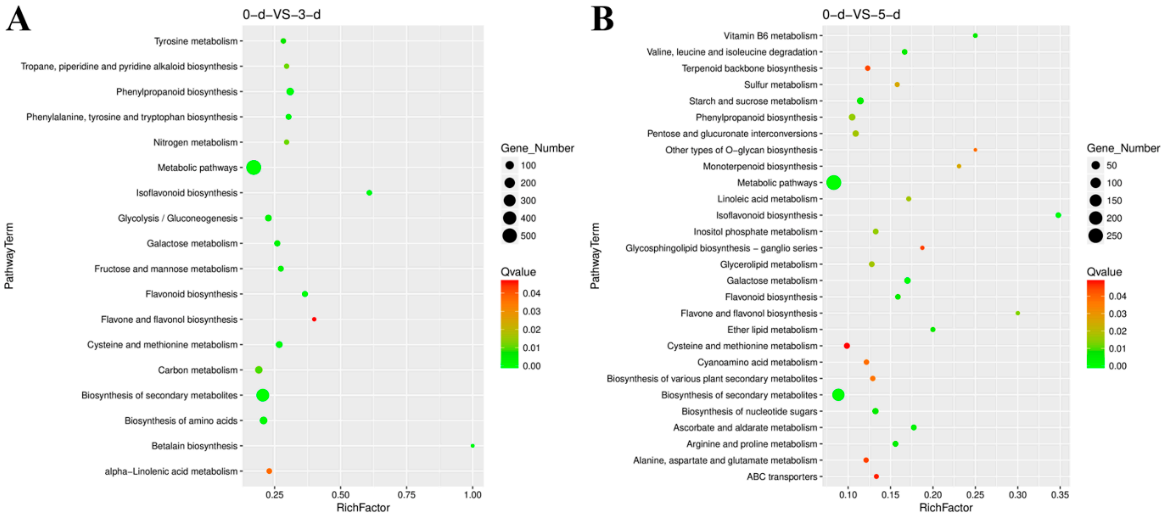

Supplementary Figure S4.

Enrichment analysis of transcriptomic data by KEGG. (A) The KEGG annotation of DEGs in HRs at 3 d post-MeJA treatment. (B) The KEGG annotation of DEGs in HRs at 5 d post-MeJA treatment.

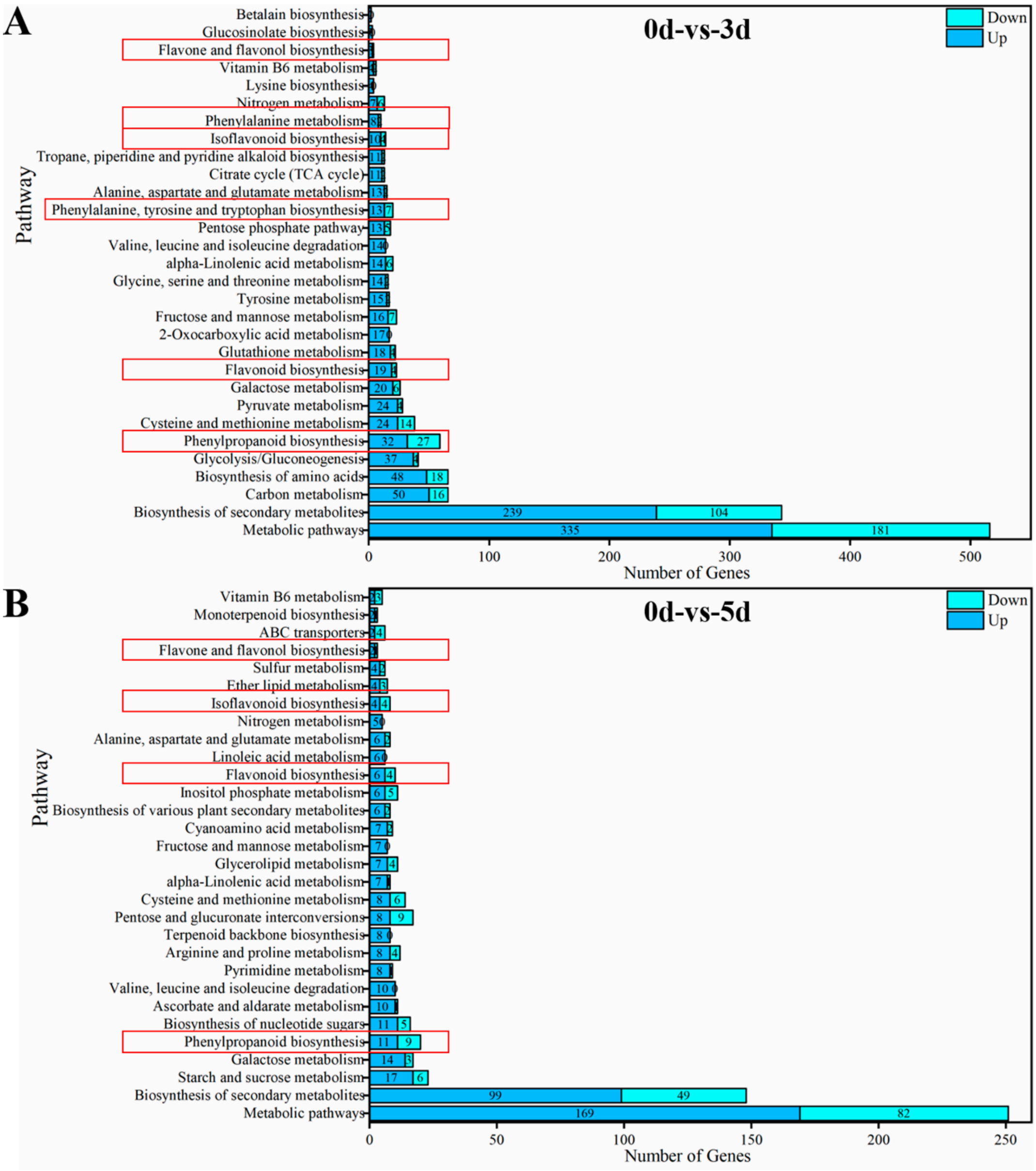

Supplementary Figure S5.

(A) After 3 days of treatment with MeJA, the metabolic pathways were enriched by up-regulated and down-regulated genes. **(B)** After 5 days of treatment with MeJA, the metabolic pathways were enriched by up-regulated and down-regulated genes. The pathways related to the synthesis of flavonoids are marked with red boxes.

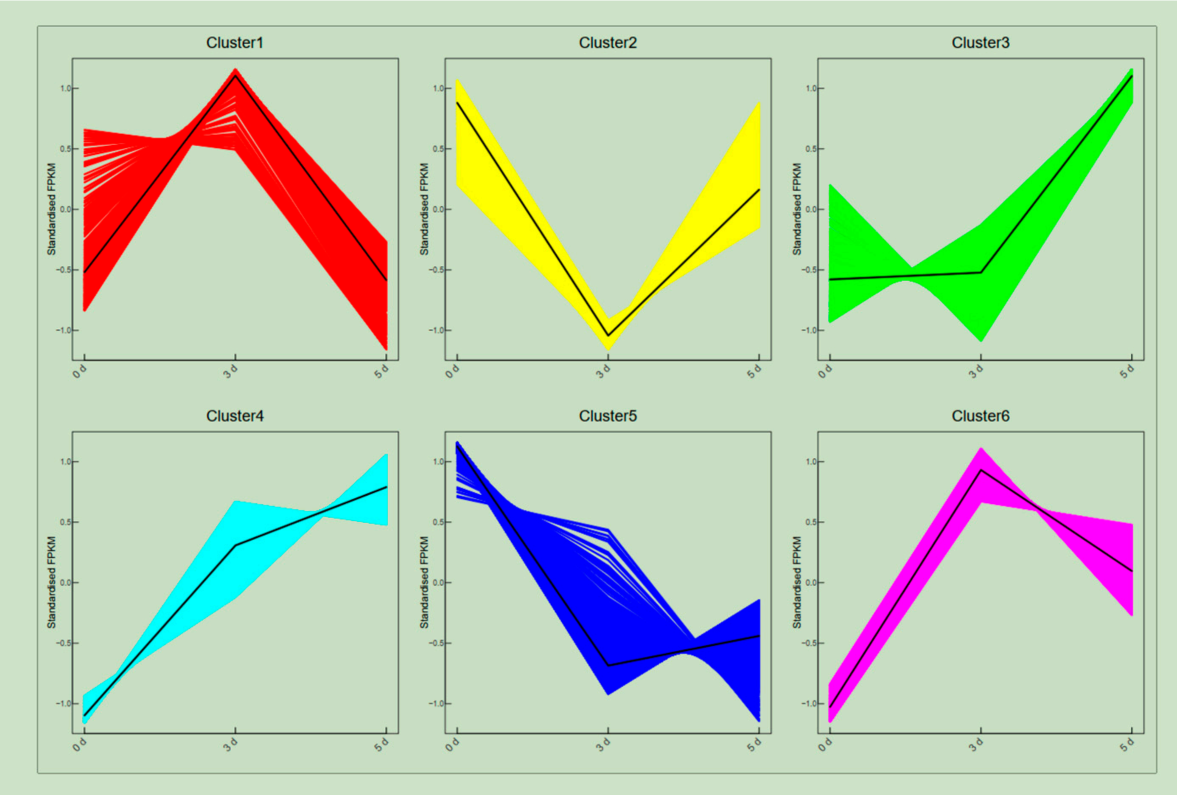

Supplementary Figure S6.

K-means clustering of gene expression profiles in all samples, resulting in six clusters with characteristic patterns.

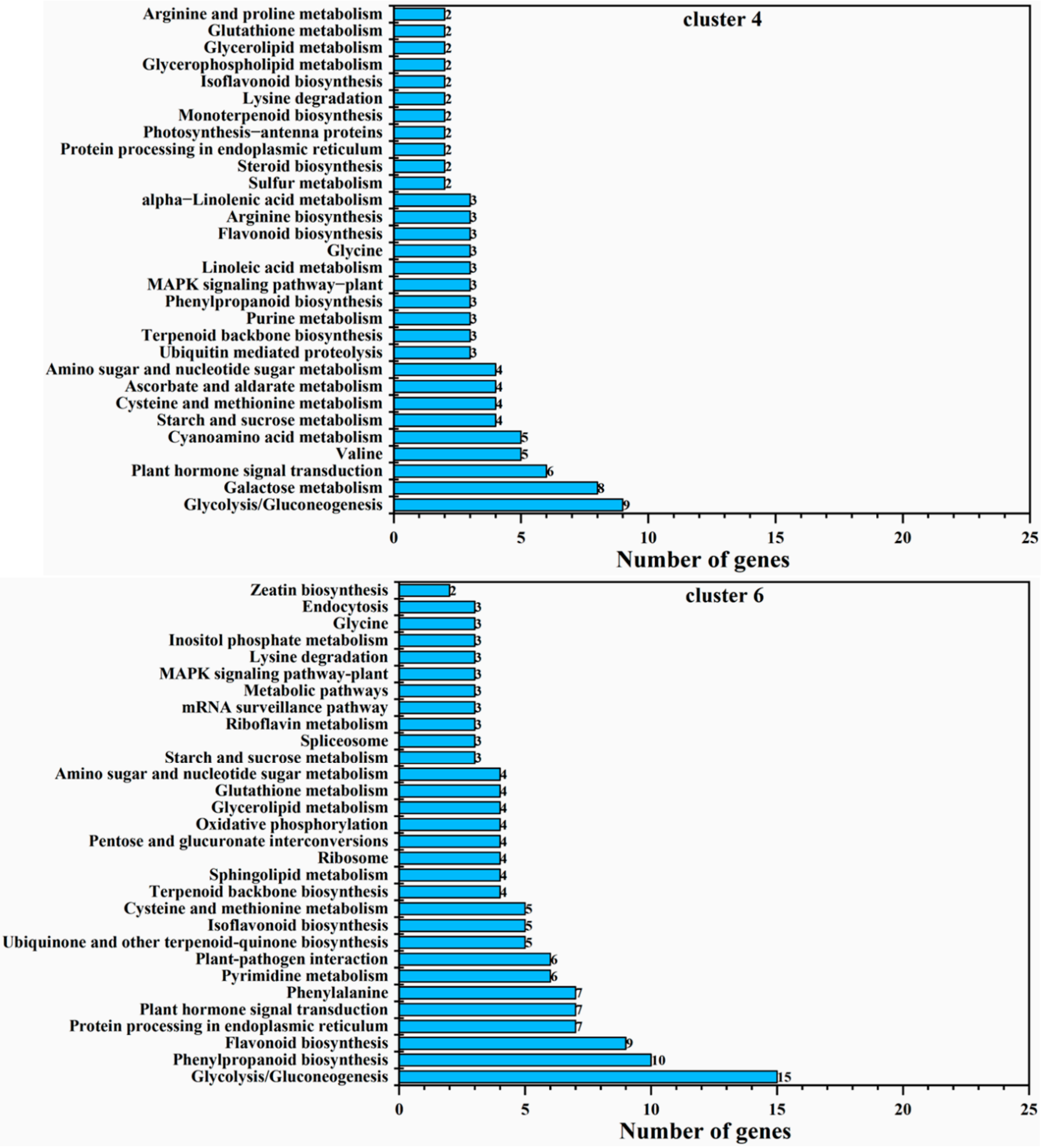

Supplementary Figure S7.

KEGG enrichment analysis of differentially expressed genes in cluster 4 and 6.

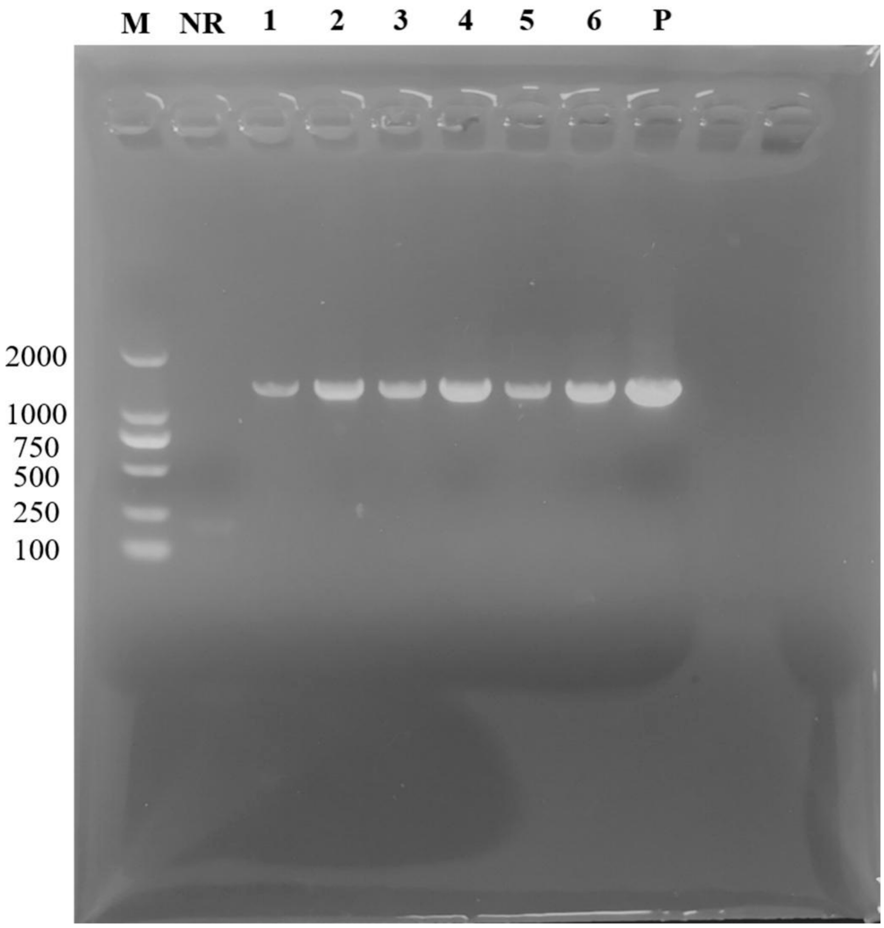

Supplementary Figure S8.

Identification of *GgCHS6*-overexpressing hairy roots by PCR. M: marker; NR: The normal root of licorice (negative control). 1-6: Different hairy root samples. P:1302-*GgCHS6* plasmid (positive control).

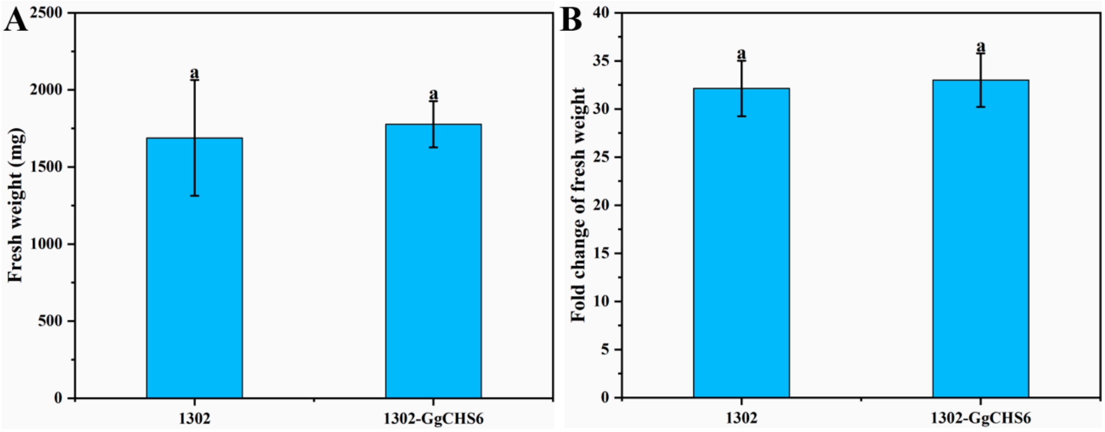

Supplementary Figure S9.

(A) The fresh weight of 1302 (empty vector) hairy roots and *GgCHS6*-overexpressing hairy roots at 28 days. **(B)** The fold increase in fresh weight of wild-type hairy roots and *GgCHS6*-overexpressing hairy roots at 28 days. Different small letters in the figure showed a significant difference (p<0.05).
