# Supplementary material for: MeJA Elicitation on Flavonoid Biosynthesis and Gene Expression in the Hairy Roots of Glycyrrhiza glabra L
Source: Genes (Basel). 2025 Nov 18;16(11):1387. doi: 10.3390/genes16111387 (PMC12652217; doi:10.3390/genes16111387)
Supplement: Supplementary file 1 [file genes-16-01387-s001.zip › Supplementary Files.docx]

Supplementary Files

MeJA elicitation on flavonoid biosynthesis and gene expression in the hairy roots of *Glycyrrhiza glabra* L.

Yutao Zhu^1*^, Bohan Wang^1^, Bingyi Xue^1^, Runqian Wang^1^, Ganlin Tang^1^, Tao Zhu^1^, Mei Zhao^1^, Taotao Li^1^, Chunli Liao^1^, Huamin Zhang^1^, Dongxiao Liu^1^, Jianhua Chen^2^, and Lianzhe Wang^1*^

^1^ College of Life Science and Engineering, Henan University of Urban Construction, Pingdingshan, 467036, China

^2^ Pingdingshan Academy of Agricultural Sciences, Pingdingshan, 467003, China

*** Correspondence:** Yutao Zhu, [zyt17494@163.com](mailto:zyt17494@163.com); Lianzhe Wang, jijy99@126.com

# Supplementary Figures and Tables

## Supplementary Figures


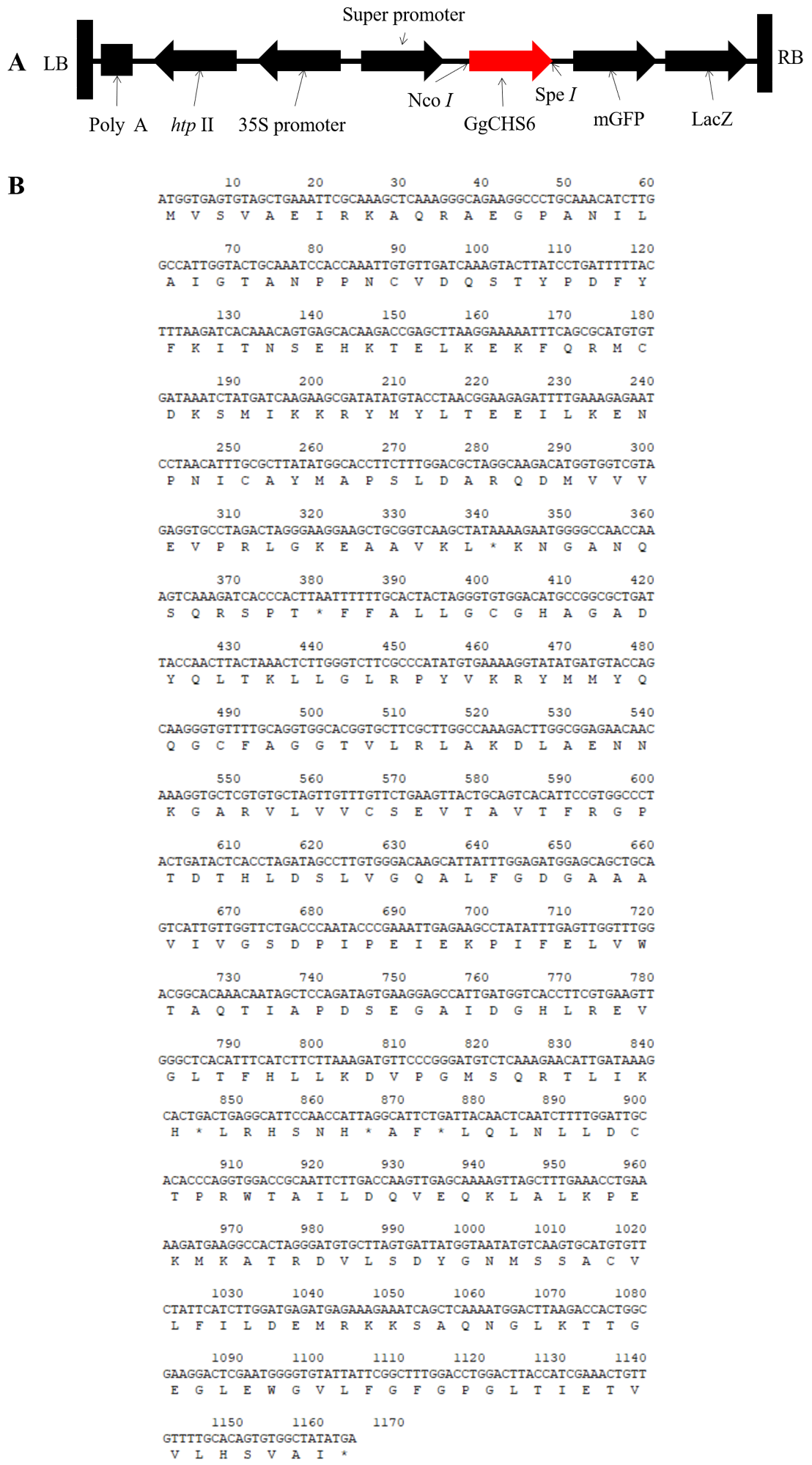
**Supplementary Figure S1.**

Construction of *GgCHS6* overexpression vector. **(A)** Schematic diagram of *GgCHS6* overexpression vector. **(B)** The DNA sequence of the *GgCHS6* and the sequence of the encoded protein.


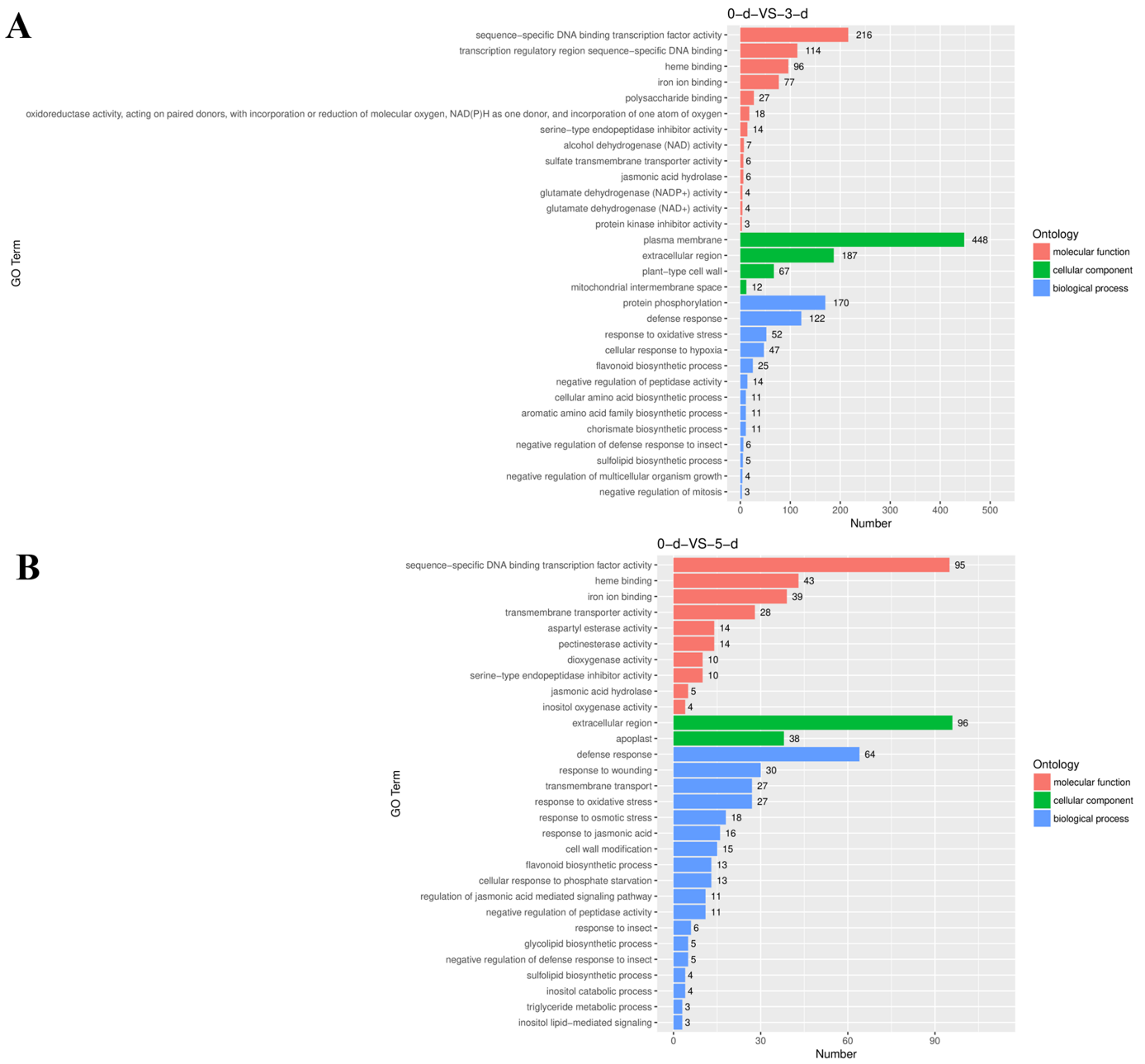


**Supplementary Figure S2.**

Gene Ontology (GO) functional classification analysis of DEGs in 0 vs. 3 d (A) and 0 vs. 5 d (B) based on RNA-Seq data. Based on sequence homology, all differentially expressed genes could be categorized into three main categories: molecular function, cellular component, and biological process.


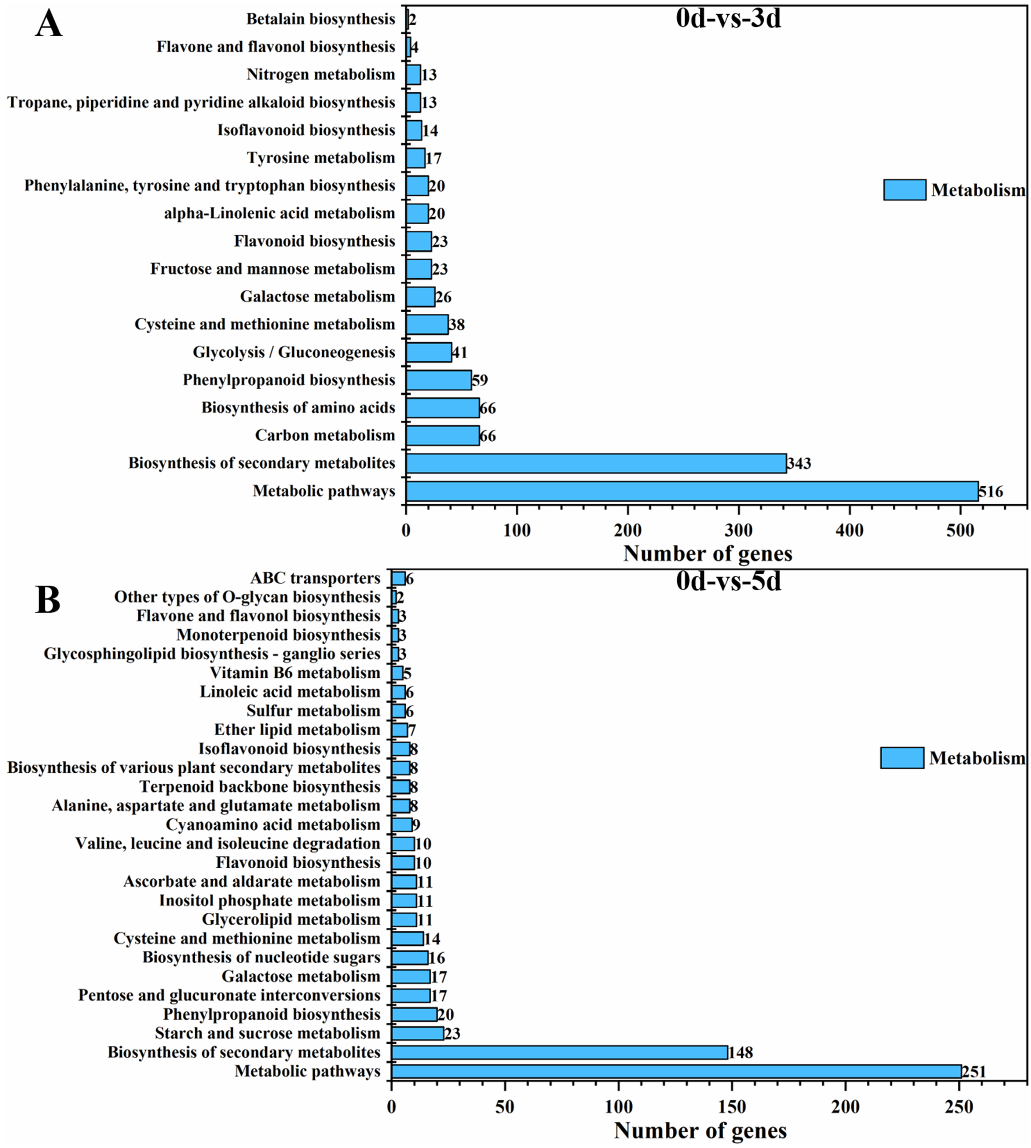


**Supplementary Figure S3.**

After 3 d and 5 d of MeJA treatment, the following KEGG pathways were mostly enriched in “metabolic pathways (ko01100)” (516 DEGs at 3 d and 251 DEGs at 5 d, respectively) and “biosynthesis of secondary metabolites (ko01110)” (343 DEGs at 3 d and 148 DEGs at 5 d, respectively).


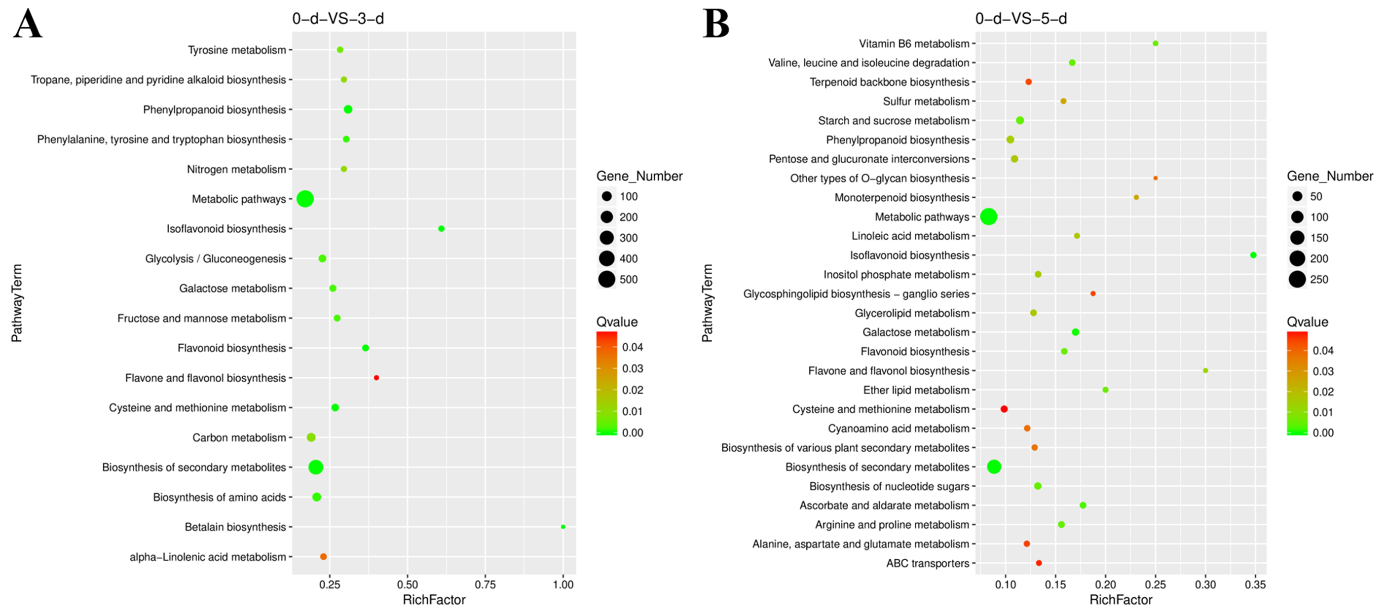
**Supplementary Figure S4.**

Enrichment analysis of transcriptomic data by KEGG. (A) The KEGG annotation of DEGs in HRs at 3 d post-MeJA treatment. (B) The KEGG annotation of DEGs in HRs at 5 d post-MeJA treatment.


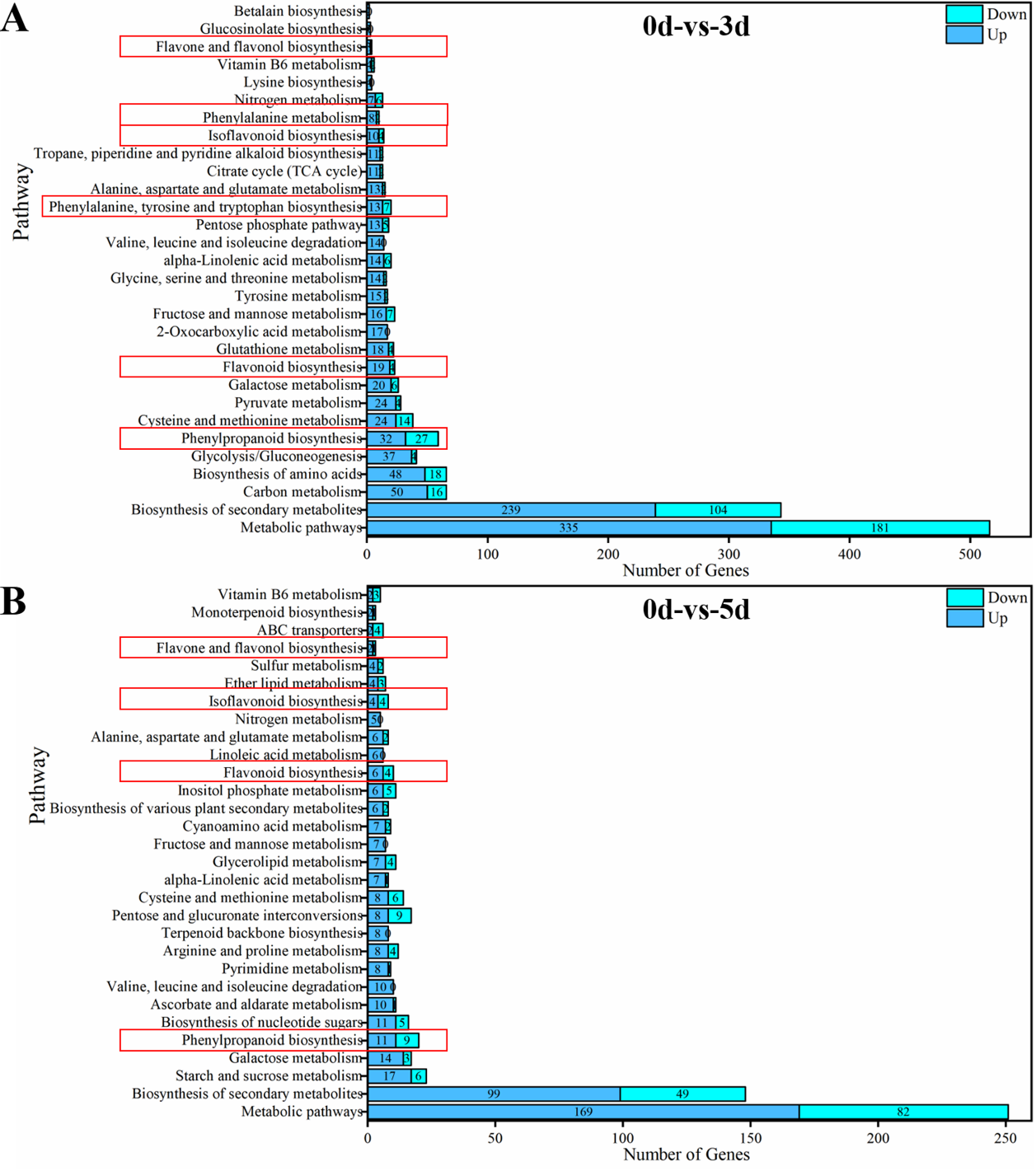


**Supplementary Figure S5.**

**(A)** After 3 days of treatment with MeJA, the metabolic pathways were enriched by up-regulated and down-regulated genes. **(B)** After 5 days of treatment with MeJA, the metabolic pathways were enriched by up-regulated and down-regulated genes. The pathways related to the synthesis of flavonoids are marked with red boxes.


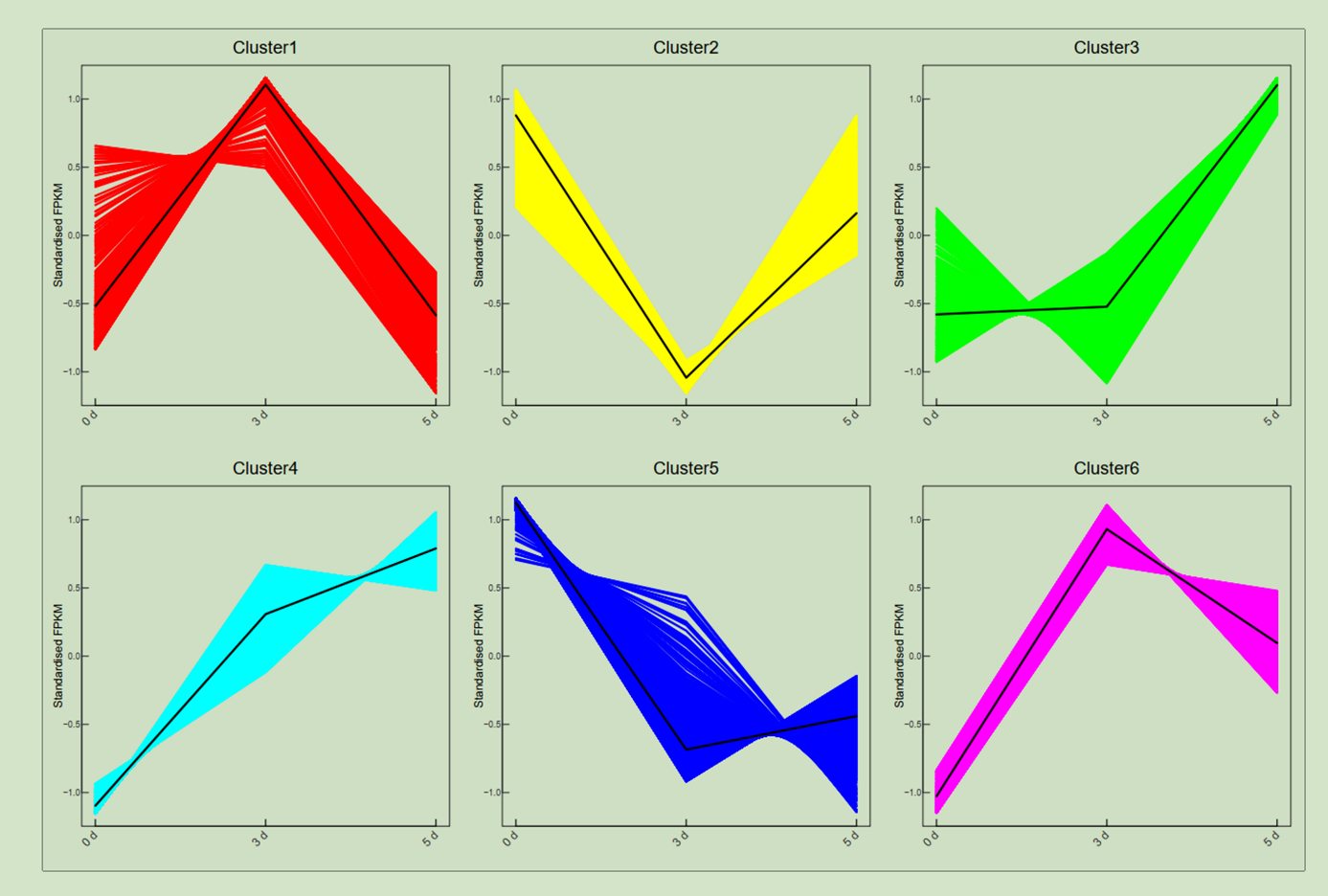
**Supplementary Figure S6.**

K-means clustering of gene expression profiles in all samples, resulting in six clusters with characteristic patterns.


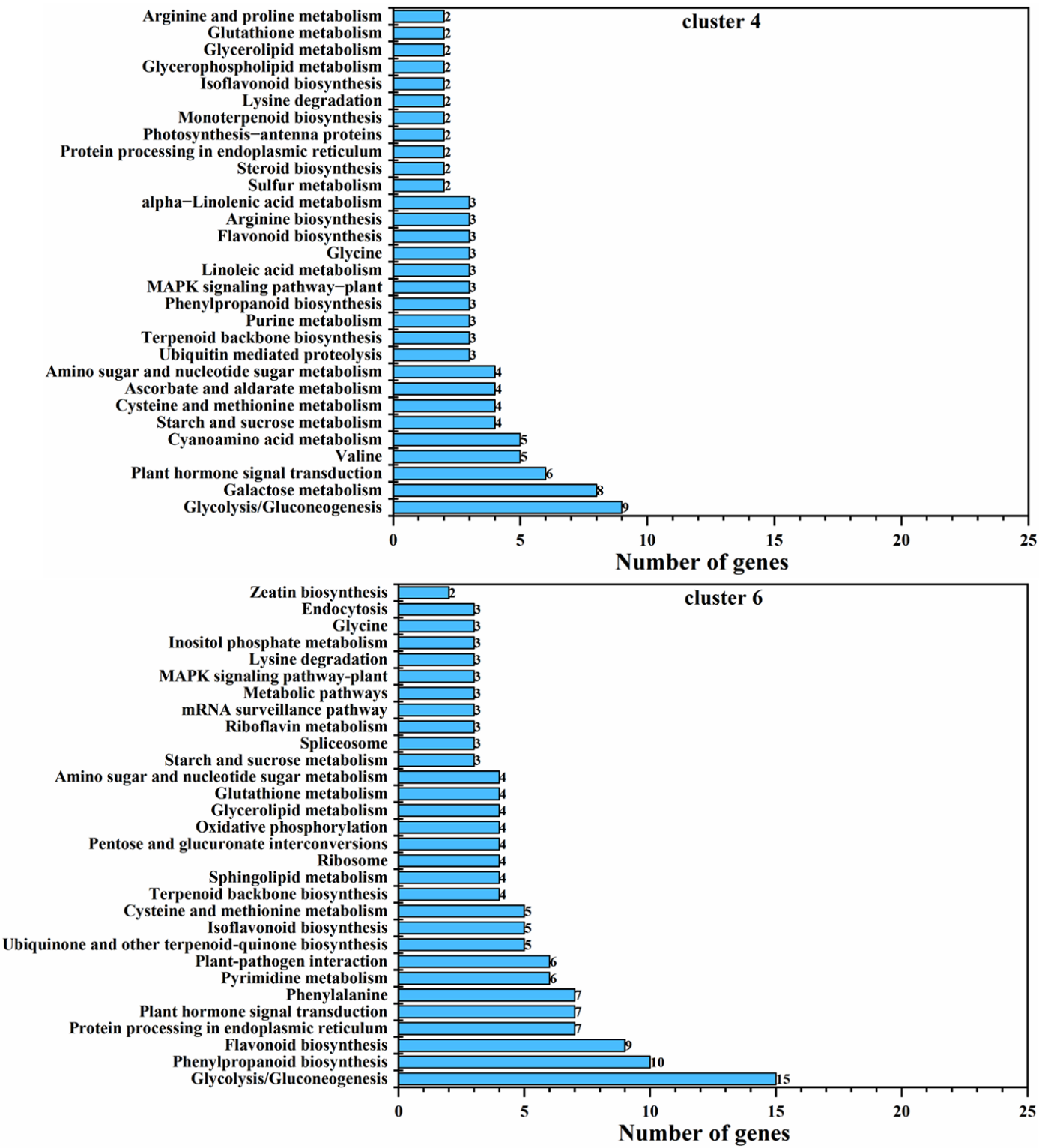
**Supplementary Figure S7.**

KEGG enrichment analysis of differentially expressed genes in cluster 4 and 6.


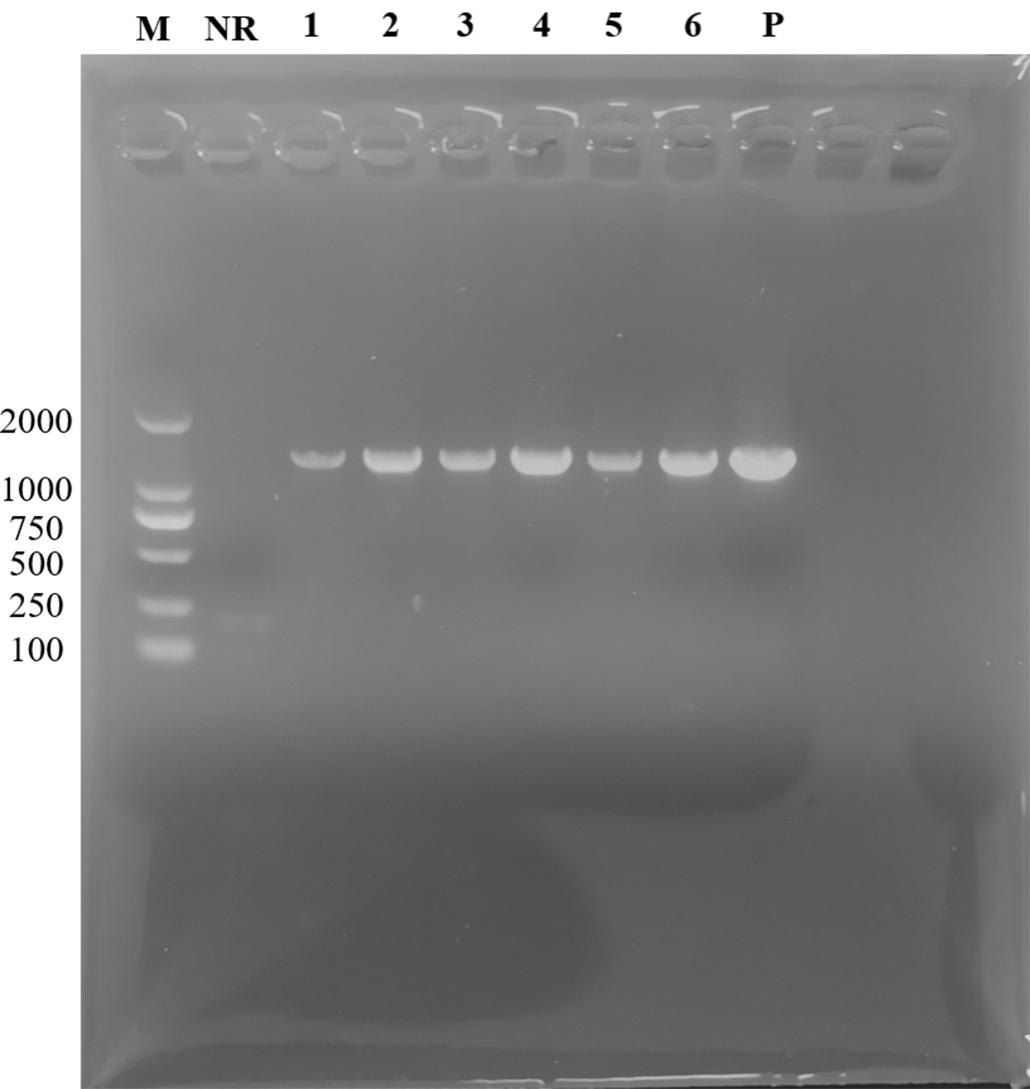


**Supplementary Figure S8.**

Identification of *GgCHS6*-overexpressing hairy roots by PCR. M: marker; NR: The normal root of licorice (negative control). 1-6: Different hairy root samples. P:1302-*GgCHS6* plasmid (positive control).


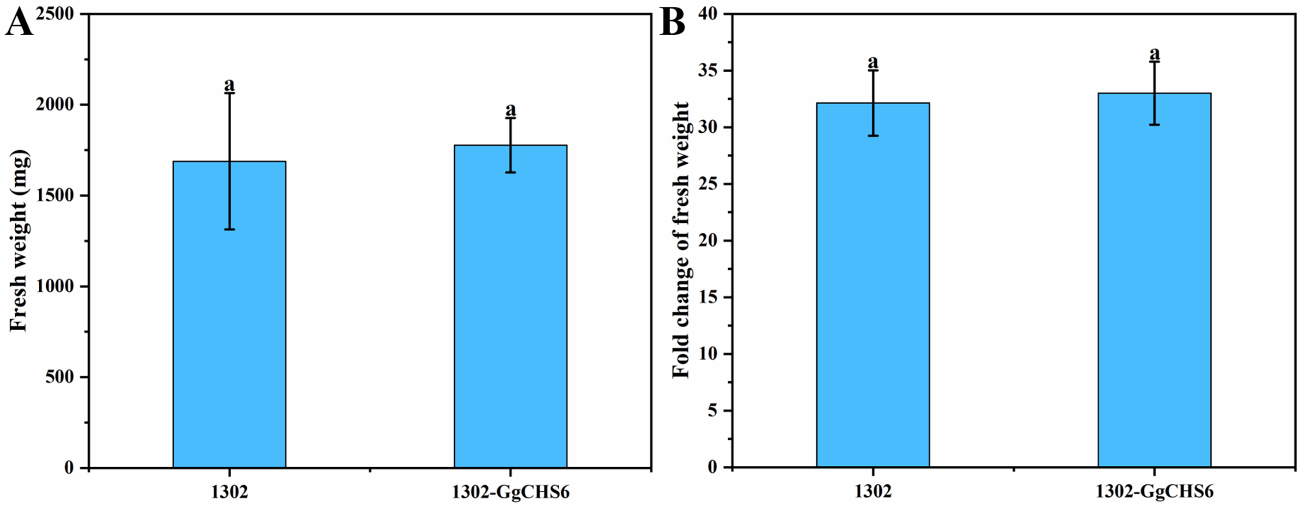


**Supplementary Figure S9.**

**(A)** The fresh weight of 1302 (empty vector) hairy roots and *GgCHS6*-overexpressing hairy roots at 28 days. **(B)** The fold increase in fresh weight of wild-type hairy roots and *GgCHS6*-overexpressing hairy roots at 28 days. Different small letters in the figure showed a significant difference (*p*<0.05).
