# Supplementary figures and images for: MeJA Elicitation on Flavonoid Biosynthesis and Gene Expression in the Hairy Roots of Glycyrrhiza glabra L
Source: Genes (Basel). 2025 Nov 18;16(11):1387. doi: 10.3390/genes16111387 (PMC12652217; doi:10.3390/genes16111387)

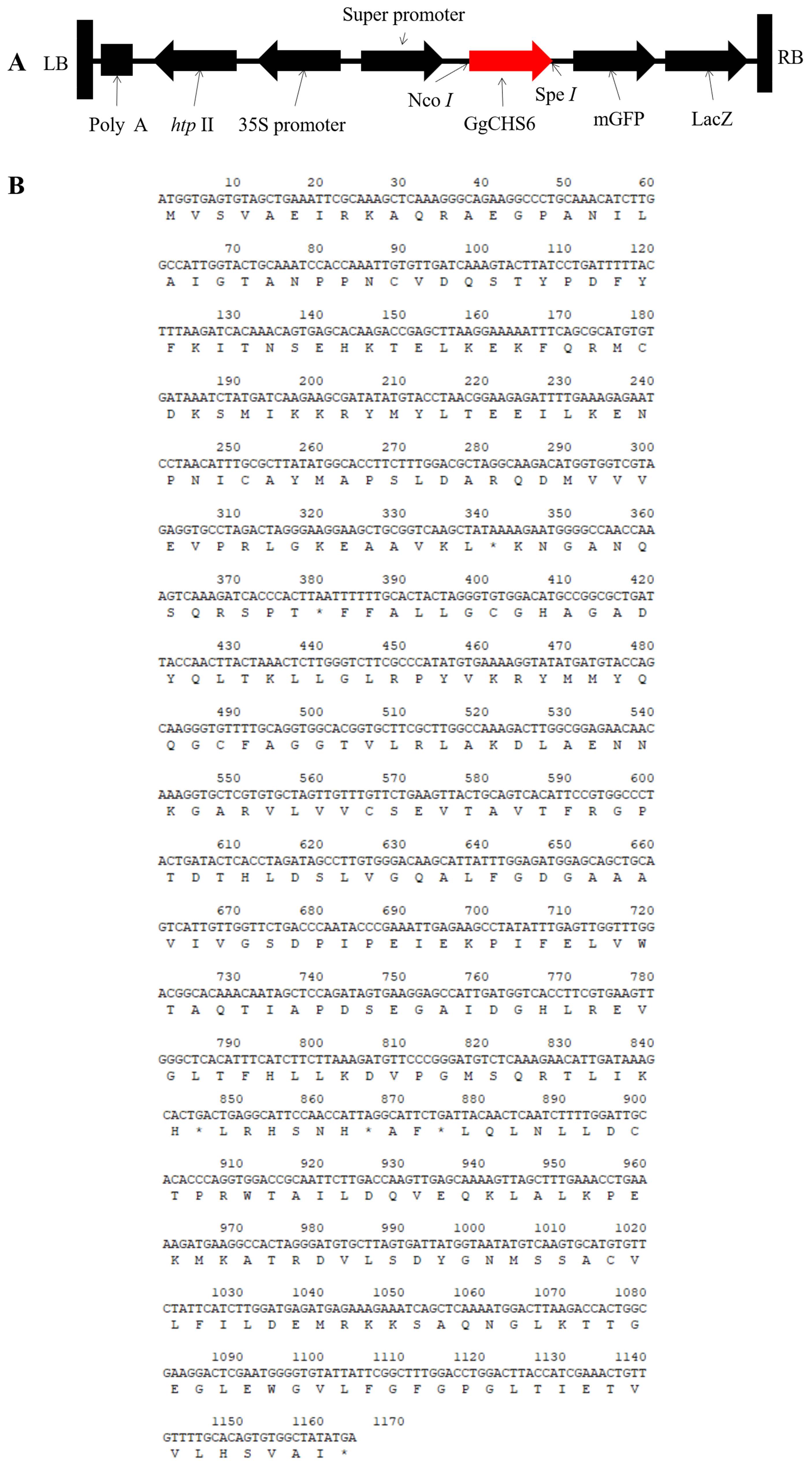

Supplement: Supplementary file 1 [file genes-16-01387-s001.zip › Figure S1.tif]

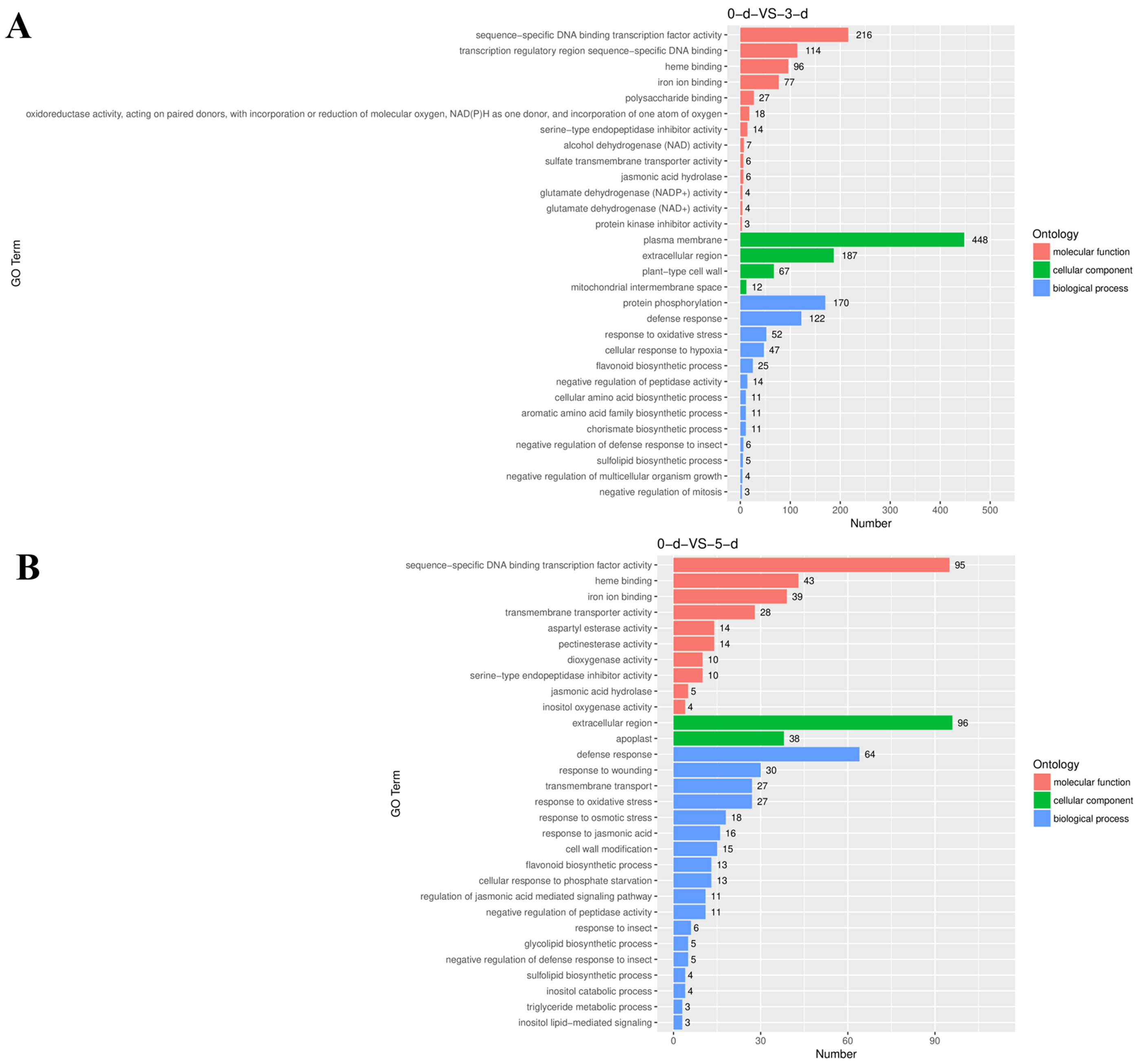

Supplement: Supplementary file 1 [file genes-16-01387-s001.zip › Figure S2.tif]

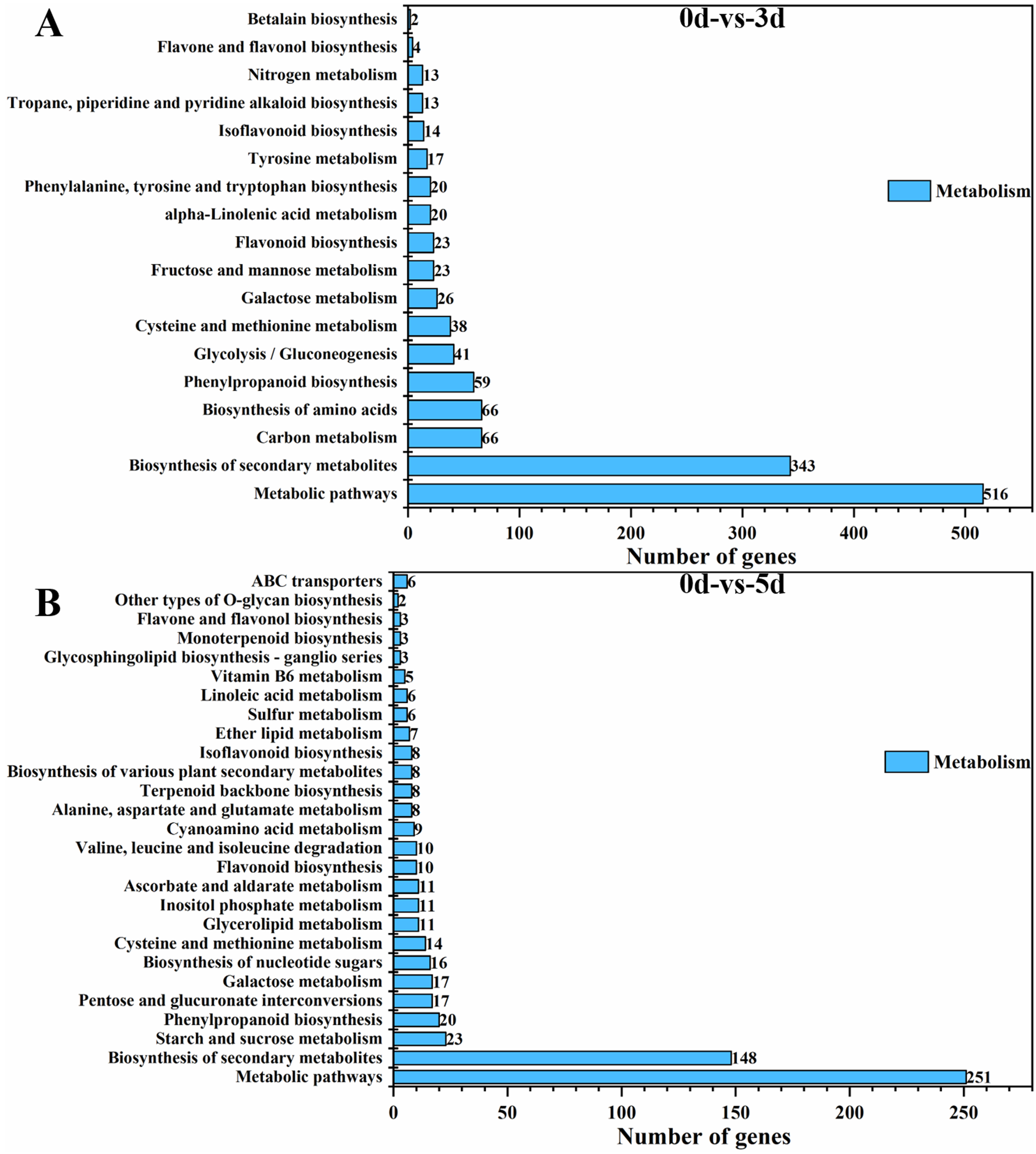

Supplement: Supplementary file 1 [file genes-16-01387-s001.zip › Figure S3.tif]

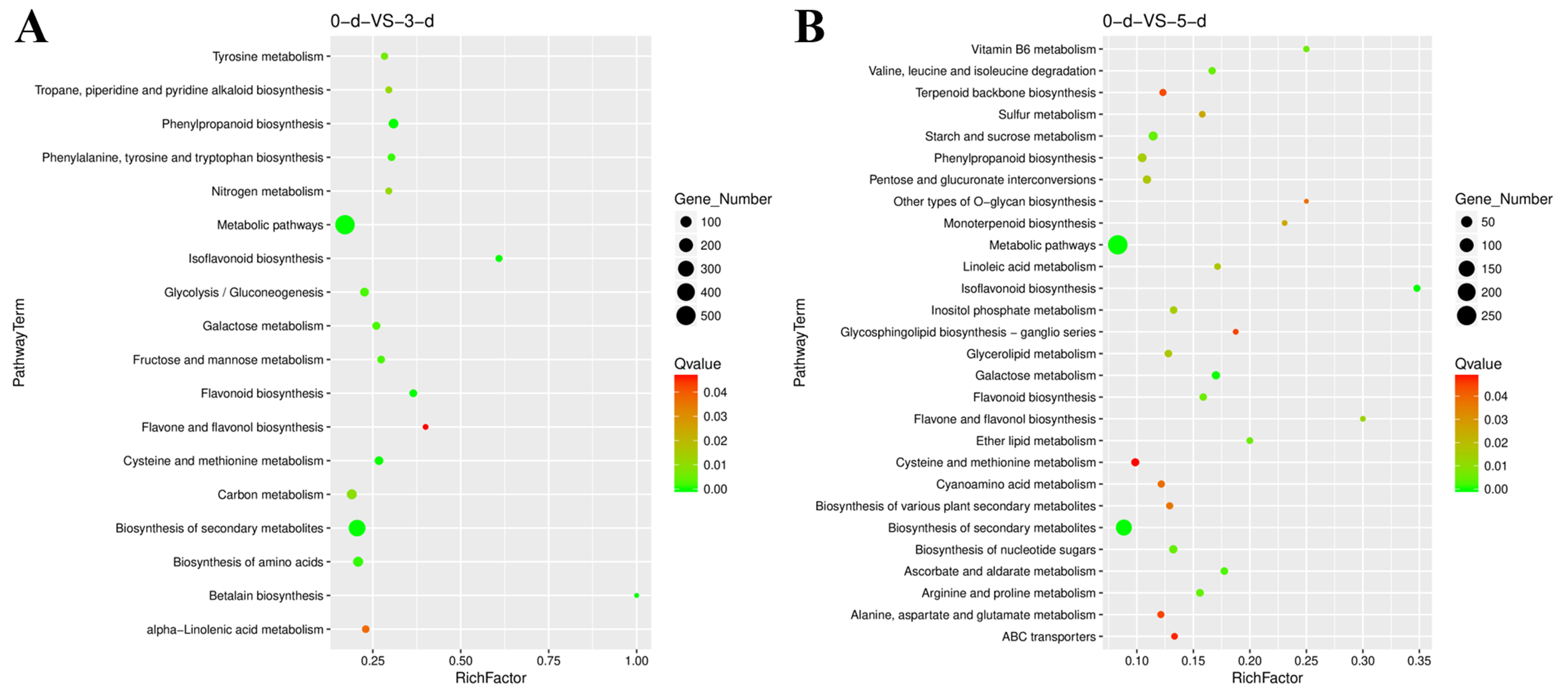

Supplement: Supplementary file 1 [file genes-16-01387-s001.zip › Figure S4.tif]

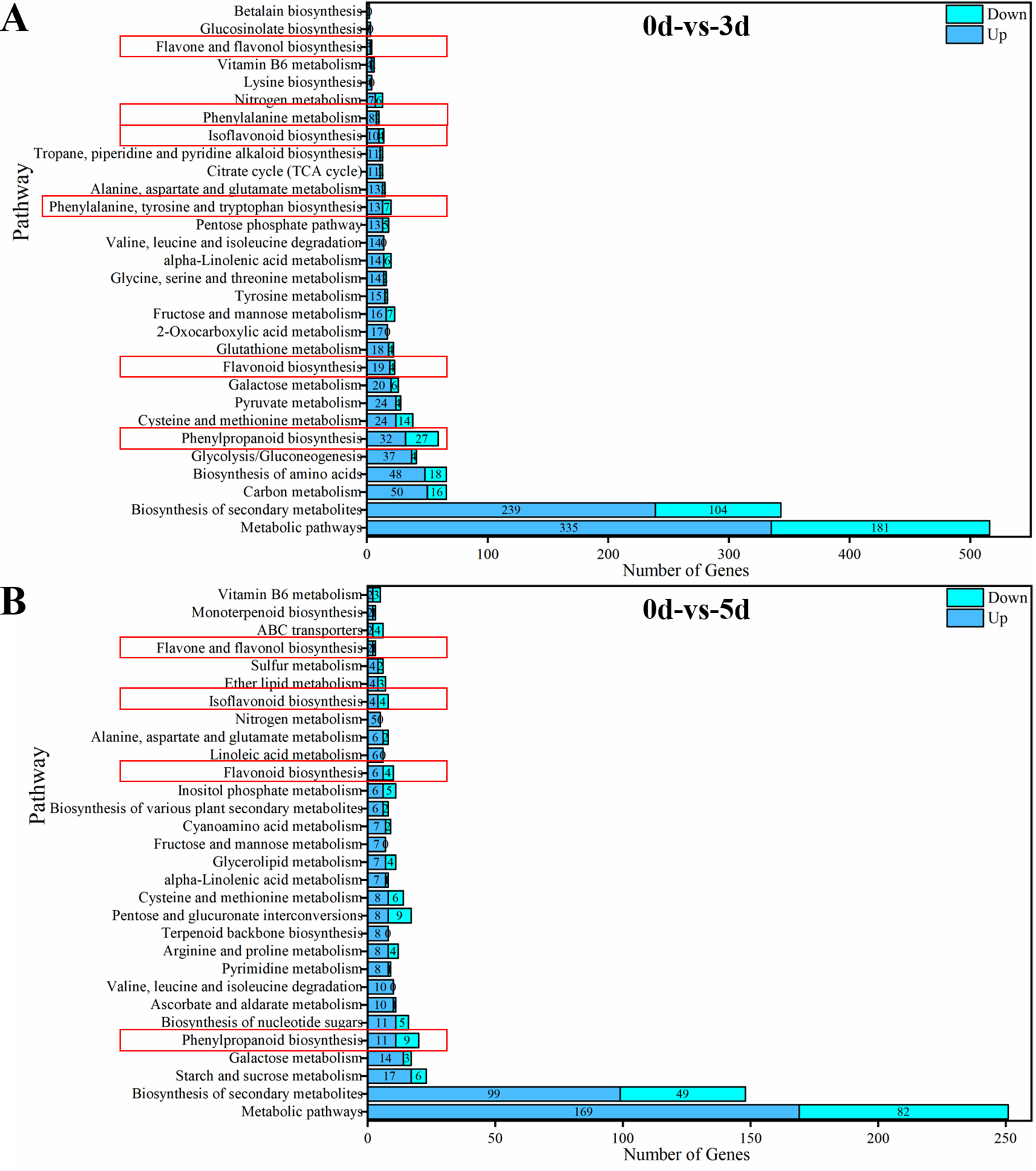

Supplement: Supplementary file 1 [file genes-16-01387-s001.zip › Figure S5.tif]

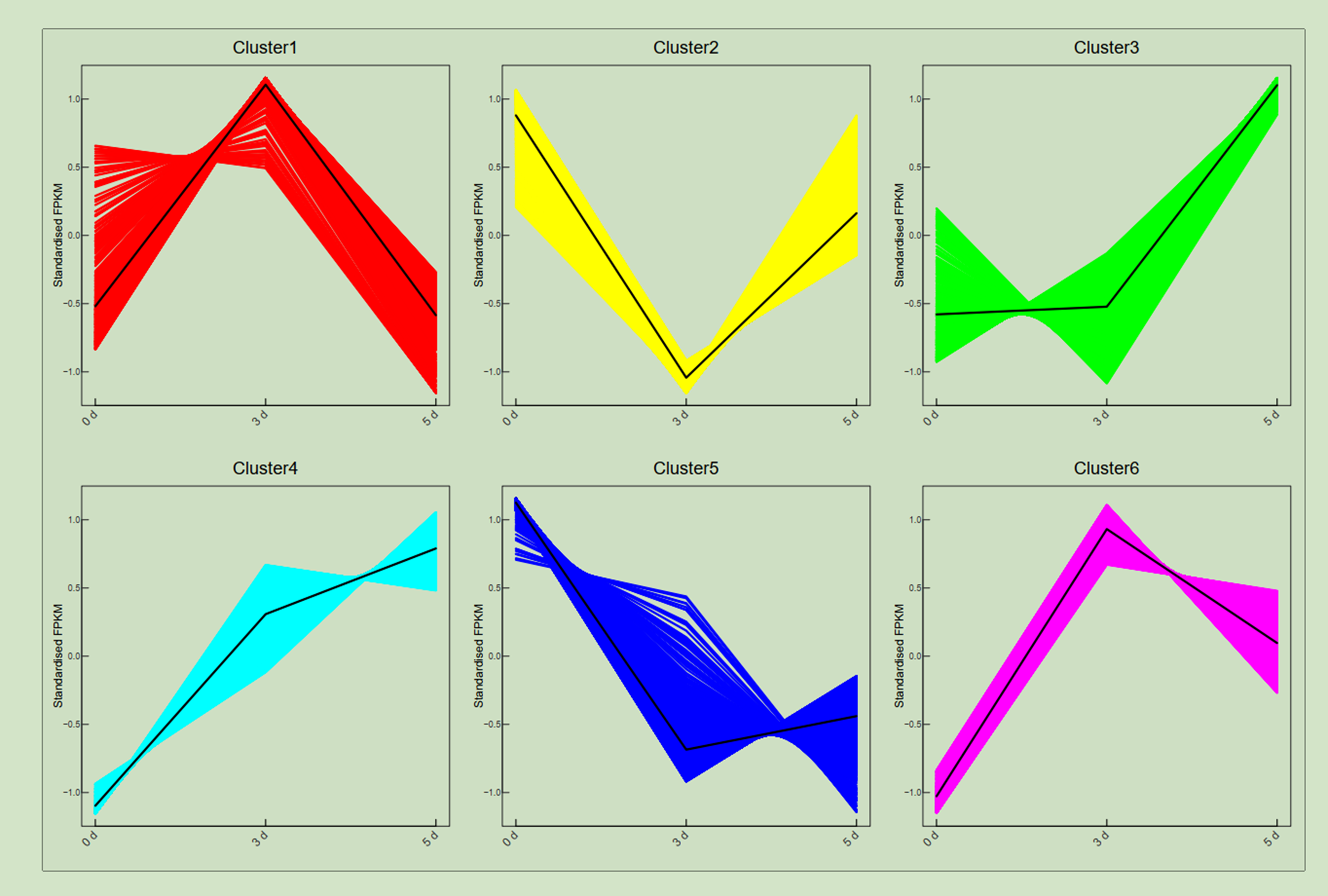

Supplement: Supplementary file 1 [file genes-16-01387-s001.zip › Figure S6.tif]

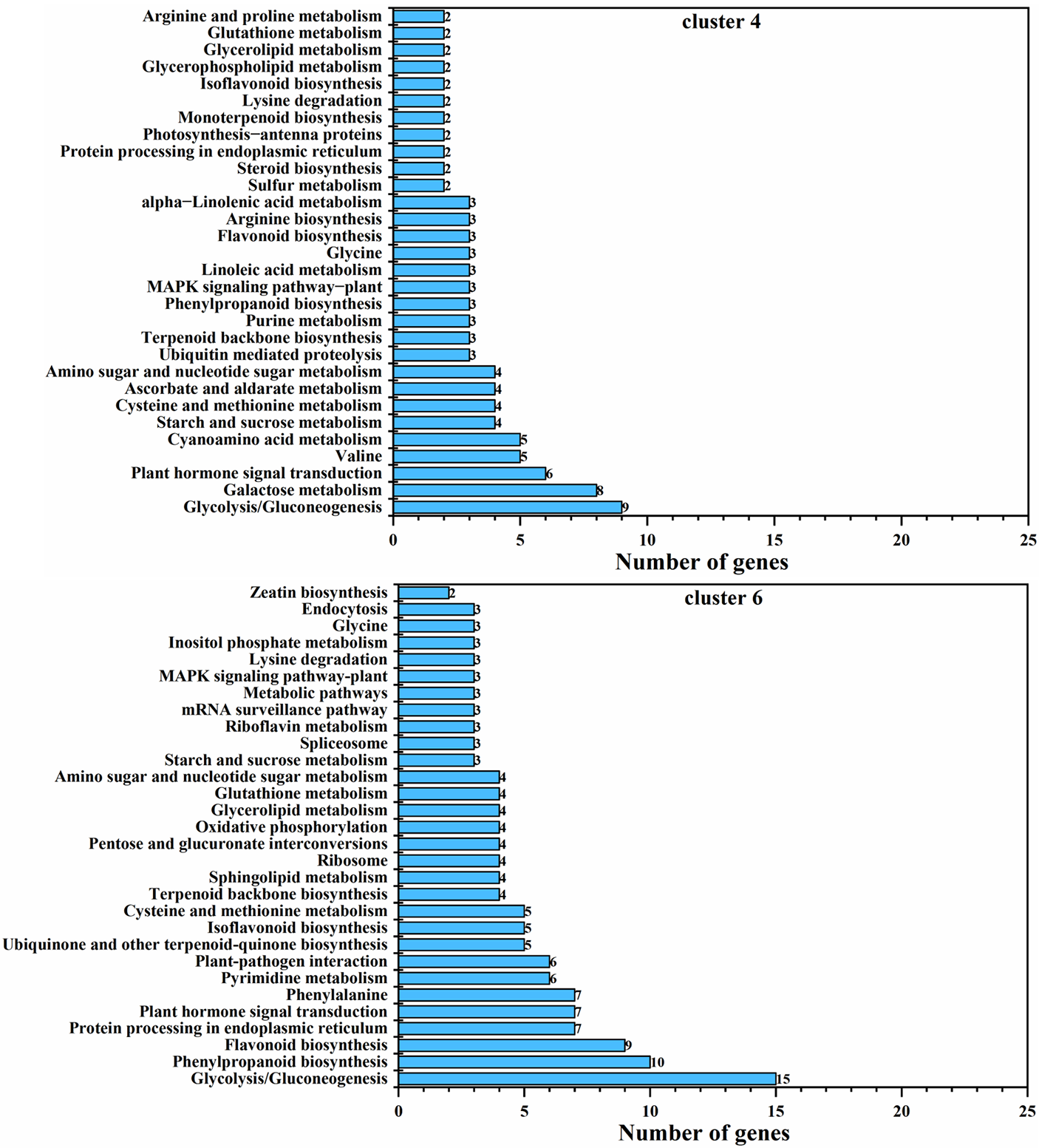

Supplement: Supplementary file 1 [file genes-16-01387-s001.zip › Figure S7.tif]

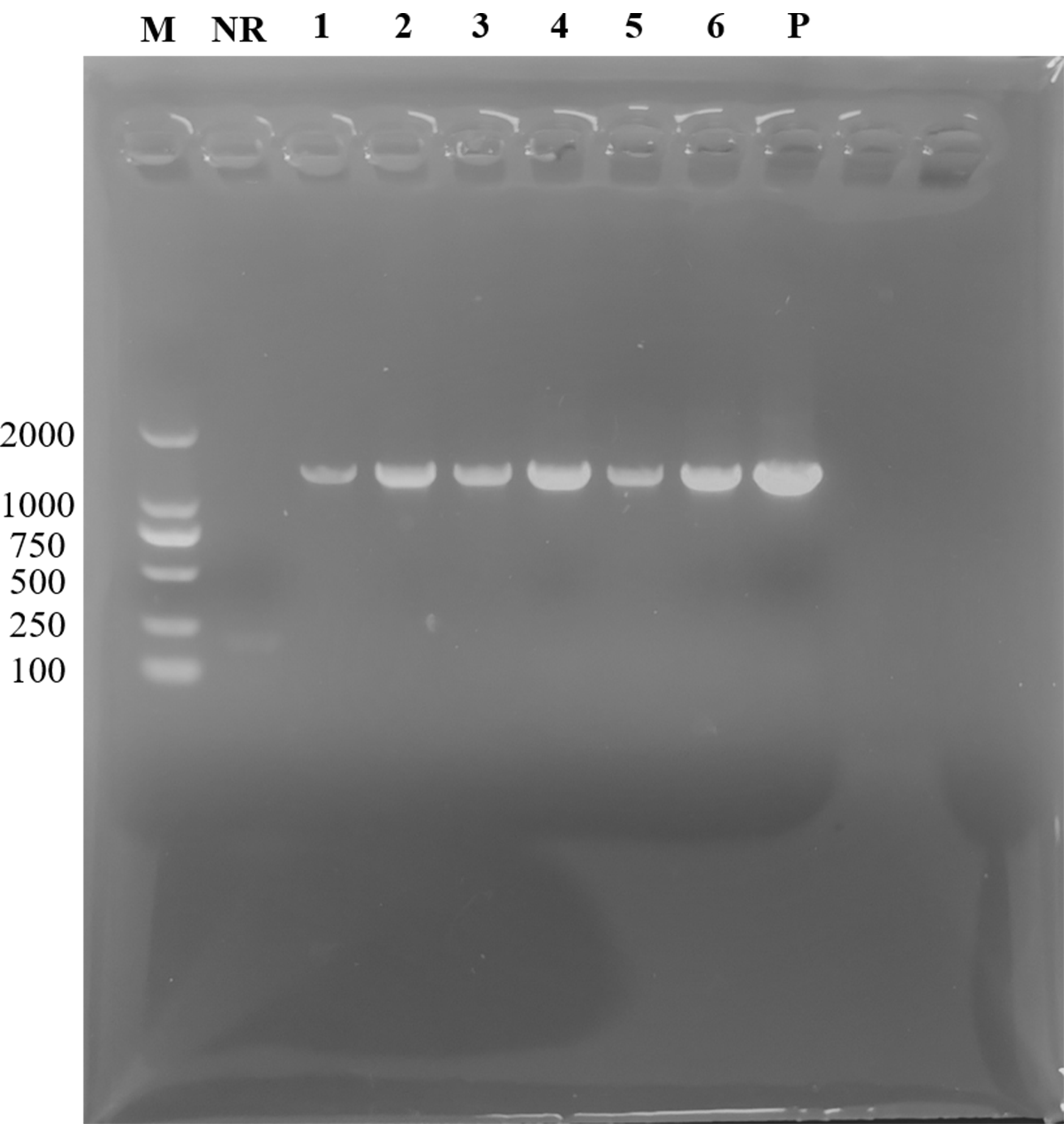

Supplement: Supplementary file 1 [file genes-16-01387-s001.zip › Figure S8.tif]

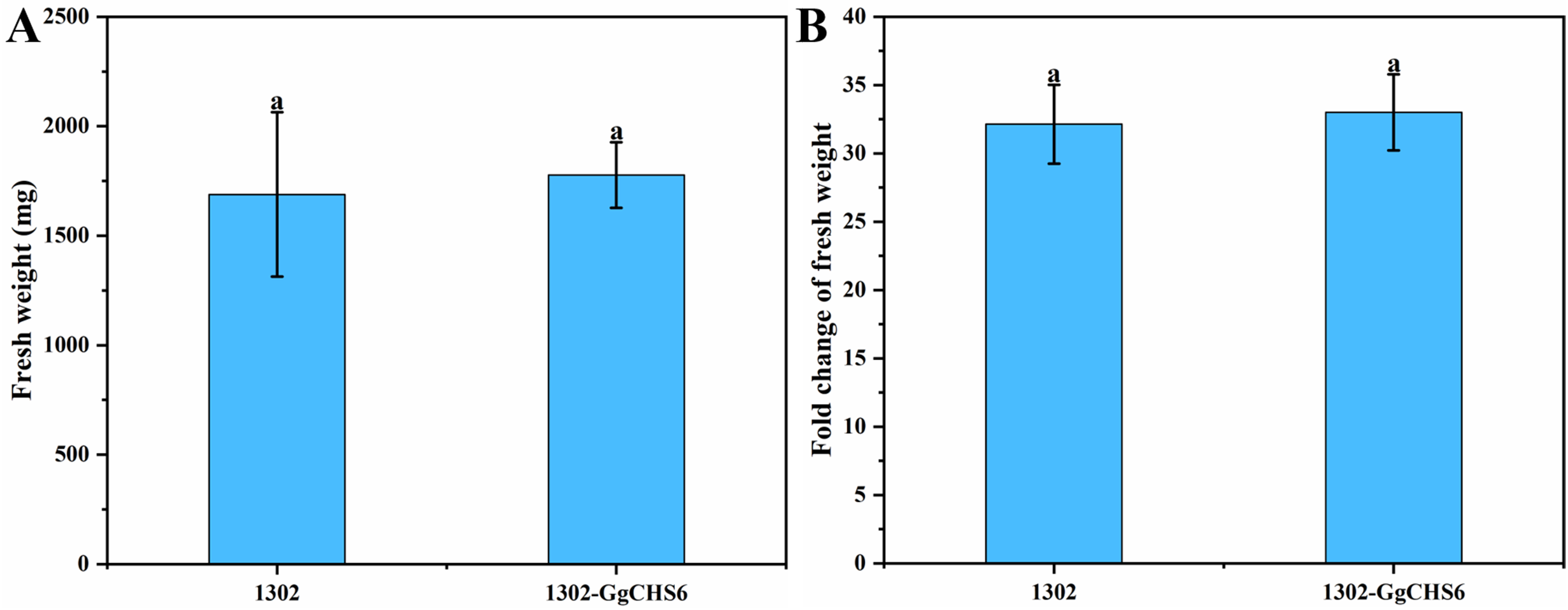

Supplement: Supplementary file 1 [file genes-16-01387-s001.zip › Figure S9.tif]
